# Nanocomposite Methacrylated Silk Fibroin-Based Scaffolds for Bone Tissue Engineering

**DOI:** 10.3390/biomimetics9040218

**Published:** 2024-04-06

**Authors:** Eugenia Spessot, Serena Passuello, Lekha Vinod Shah, Devid Maniglio, Antonella Motta

**Affiliations:** 1 Department of Industrial Engineering and BIOtech Research Centre, University of Trento, Via Sommarive 9, 38123 Trento, Italy; eugenia.spessot@unitn.it (E.S.); lekhavinod.shah@unitn.it (L.V.S.); antonella.motta@unitn.it (A.M.); 2 European Institute of Excellence on Tissue Engineering and Regenerative Medicine Unit, Via delle Regole 101, 38123 Trento, Italy

**Keywords:** methacrylated silk fibroin, hydroxyapatite, scaffold, bone tissue engineering

## Abstract

The treatment of bone defects is a clinical challenge. Bone tissue engineering is gaining interest as an alternative to current treatments, with the development of 3D porous structures (scaffolds) helpful in promoting bone regeneration by ensuring temporary functional support. In this work, methacrylated silk fibroin (SilMA) sponges were investigated as scaffolds for bone tissue engineering by exploiting the combination of physical (induced by NaCl salt during particulate leaching) and chemical crosslinking (induced by UV-light exposure) techniques. A biomimetic approach was adopted to better simulate the extracellular matrix of the bone by introducing either natural (mussel shell-derived) or synthetic-origin hydroxyapatite nanoparticles into the SilMA sponges. The obtained materials were characterized in terms of pore size, water absorption capability and mechanical properties to understand both the effect of the inclusion of the two different types of nanoparticles and the effect of the photocrosslinking. Moreover, the SilMA sponges were tested for their bioactivity and suitability for bone tissue engineering purposes by using osteosarcoma cells, studying their metabolism by an AlamarBlue assay and their morphology by scanning electron microscopy. Results indicate that photocrosslinking helps in obtaining more regular structures with bimodal pore size distributions and in enhancing the stability of the constructs in water. Moreover, the addition of naturally derived hydroxyapatite was observed to be more effective at activating osteosarcoma cell metabolism than synthetic hydroxyapatite, showing a statistically significant difference in the AlamarBlue measurement on day 7 after seeding. The methacrylated silk fibroin/hydroxyapatite nanocomposite sponges developed in this work were found to be promising tools for targeting bone regeneration with a sustainable approach.

## 1. Introduction

The treatment of bone defects represents a significant challenge in clinical settings, despite bone having a high capability for self-regeneration. Severe injuries or the removal of bone tissue beyond a critical volume can hinder the self-repair process [1]. Current treatment options, such as autografts and allografts, are hindered by limitations, including limited availability, donor site morbidity and immunogenicity risks [2].

Consequently, there is a growing focus on tissue engineering strategies aimed at harnessing the self-healing potential of bone. Bone formation typically involves two processes: intramembranous ossification, where mesenchymal stem cells directly differentiate into cells forming bone tissue, and endochondral ossification, where a cartilaginous intermediate (callus) is formed and subsequently mineralized before turning into bone [3]. While existing tissue engineering approaches primarily focus on intramembranous ossification, most bone regeneration in nature occurs through endochondral ossification, a process not adequately mimicked by engineered scaffolds [4]. Mimicking the endochondral mechanism holds potential for developing advanced bone substitutes that can effectively heal large bone fractures [5,6].

Central to these strategies is the use of porous, three-dimensional structures, commonly referred to as scaffolds, which serve to temporarily fill the bone defect, hence facilitating cell colonisation and extracellular matrix (ECM) production, before subsequently degrading within the body.

The design of these scaffolds is crucial, with specific features such as porosity, water uptake, mechanical properties and biological compatibility, carefully tailored to recreate an environment suitable for cell activity such as cell adhesion, infiltration, proliferation and differentiation [7]. Scaffold structures with tuneable properties can be engineered to suit the requirements of the bone microenvironment by selecting appropriate biomaterials, architectures and bioactive cues [8]. Recent advancements in the field have seen a shift from ‘hard’ to ‘soft’ materials in the attempt to provide suitable support for bone tissue, exploiting adjustments in the mechanical properties, the use of different fabrication methods, and effective delivery of bioactive molecules [9]. Achieving a precise mimicry of the bone microenvironment involves a combination of cells, soft matrices and inorganic particulates to support regenerative processes and facilitate molecular signalling. Natural-derived materials, when organised into sponges or hydrogels, show promise in promoting bone regeneration [10,11,12,13,14,15]. Silk fibroin (SF), a globular protein derived from silkworms or spiders, has gained significant attention in tissue engineering due to its unique properties and it has already been approved by the FDA for specific clinical applications [16,17]. The main properties are the ease of chemical functionalization and bioconjugation, high processability, cytocompatibility and tunability of mechanical properties based on fabrication mechanisms and parameters [18,19,20,21]. Notably, SF-based sponges have been extensively used in bone tissue engineering, having shown their ability to promote bone formation both in vitro and in vivo [22,23,24,25,26,27]. However, the fabrication methods of these sponges involved the formation of a physically crosslinked hydrogel network to stabilise their structure, which typically implies a change in the protein secondary structure from random coil to beta-sheet. Physical crosslinking is usually less stable than chemical cross-linking, where stability is guaranteed by the formation of strong chemical bonds in a 3D network. Thanks to the ease of functionalization of SF, it is possible to turn it into a UV-photocrosslinkable material through methacrylation reactions. Glycidyl methacrylate (GMA) can be used to modify the amine side groups on the lysine present in the SF primary structure, to obtain aqueous solutions of methacrylated silk fibroin (SilMA) [28]. SilMA has been widely used as a starting material for different architectures aimed at tissue engineering purposes, such as bioinks [29], hydrogels [30,31] and membranes [32,33]. In a previous study, the combination of physical (cryo-gelation) and chemical (ethylene glycol diglycidyl ether) crosslinking in SF sponges conferred them with a wider range of physico-chemical properties, making them suitable for a wide variety of applications in tissue engineering [34]. However, SilMA sponges were fabricated by exploiting only UV-chemical crosslinking [35]. To increase the potential of SilMA-based scaffolds for bone tissue engineering, we have attempted for the first time to combine physical (induced by NaCl salt during particulate leaching) and chemical crosslinking (induced by UV-light exposure) on SilMA sponges.

Furthermore, the incorporation of bioactive factors, such as inorganic particles, into SF- or SilMA-based scaffolds holds potential for enhancing biological activity, including osteoconductivity and osteoinductivity, thereby enhancing the bone regenerative potential of the material [36]. In fact, the inclusion of bioactive glass particles [37], magnetite nanoparticles [38,39] and calcium phosphate particles [40,41,42] in SF-based scaffolds has enhanced biological compatibility for bone tissue regeneration.

In particular, hydroxyapatite (HAP) has been widely employed in SF-based scaffolds to mimic bone tissue due to its similarity with bone apatite, which shows promising results for bone tissue regeneration both in vivo and in vitro [41,43,44,45,46]. HAP can be obtained either by synthetic routes or by extraction from natural sources, such as mussel shells, fish bones, and eggshells. Natural-derived HAP is usually enriched with trace ions or organic components, offering advantages for bone tissue regeneration [47,48]. Moreover, their use contributes to the development of more sustainable biomaterials in line with the principles of circular economy, which involve the reuse of food waste [49]. 

Previously, eggshell-derived HAP particles at a concentration of 1–2 wt% in injectable SF-hydrogels for alveolar bone showed improved biological activity compared to the synthetic one [50]. Also, mussel shell-derived HAP scaffolds were observed to confer higher cellular compatibility for hMSCs than synthetic HAP [51]. However, the use of mussel shell-derived HAP included in SilMA-based sponges has not been reported in the literature yet.

In this study, firstly, methacrylated silk fibroin (SilMA) sponges were investigated as scaffolds for bone tissue engineering by exploiting a combination of physical and chemical crosslinking, with the first induced by salt grain leaching and the second by UV exposure in the presence of a photo-initiator. 

Additionally, a biomimetic approach was adopted to better simulate the ECM of the bone tissue by introducing hydroxyapatite nanoparticles, either of natural (mussel shell-derived) or synthetic origin, to the SilMA sponges. The obtained materials were extensively characterised in terms of porosity, water absorption capability and mechanical properties to understand both the effect of the inclusion of HAP of different origins and the effect of photocrosslinking. Moreover, the sponges were tested for their bioactivity and suitability for bone tissue engineering purposes by using osteosarcoma cells and studying their morphology through scanning electron microscopy and their metabolism by an AlamarBlue assay. The findings indicate that certain conditions (double crosslinking and natural-derived hydroxyapatite) exhibit better potential for their application in bone tissue engineering.

## 2. Materials and Methods

### 2.1. Scaffold Preparation 

#### 2.1.1. Preparation of Methacrylated Silk Fibroin (SilMA) Solution

Silk fibroin was extracted from Bombyx mori silk cocoons (from Chul Thai Silk Co., Phetchabun, Thailand) and purified, adapting well-known protocols [18,52]. Briefly, to remove silk sericin and to extract silk fibroin, the silk cocoons were delaminated and cut into small pieces. Then, 10 g of sliced cocoons were boiled in 4 L of a 0.02 M hot sodium carbonate solution (Na_2_CO_3_, Sigma-Aldrich, St. Louis, MO, USA) for 30 min. The fibres were then rinsed using distilled water three times for 20 min and dried at room temperature for 2 days.

Further, 6 g of the degummed silk fibres were dissolved in 30 mL of 9.3 M lithium bromide (LiBr—Merck Sigma Aldrich, Darmstadt, Germany) solution at 60 °C for 3 h. 

After the fibres were completely dissolved, silk fibroin was chemically modified by carrying out a methacrylation reaction adapted from an existing procedure described elsewhere [35,52]. Briefly, 424 mM of glycidyl methacrylate (GMA—Merck Sigma Aldrich, Darmstadt, Germany) was added dropwise into the silk fibroin solution and left to react for 3 h at 60 °C under continuous stirring at 300 rpm. The mechanism of the chemical reaction is a nucleophilic addition causing the opening of the epoxy ring present in the GMA, which reacts with the primary amines present in the lysine side groups in silk fibroin, forming a di-β-hydroxyamide group [28]. The solution was dialyzed for four days against distilled water using a 3.5 kDa cutoff dialysis tube to remove the excess of LiBr and GMA. The concentration of the solution at the end of the dialysis was measured by UV spectroscopy (Nanodrop 1000, ThermoFisher Scientific, Waltham, MA, USA), evaluating the intensity of the peak at 280 nm. The SilMA solution was then concentrated to 8% *w*/*v*. The pH of the SilMA solution was measured and stored at 4 °C until further use.

#### 2.1.2. Hydroxyapatite Sources, Synthesis and Characterization

Mussel shell-derived hydroxyapatite powder (HAPm) was produced via mechano-chemical synthesis in the processing laboratory of the Department of Industrial Engineering at the University of Trento, as previously reported [51]. Briefly, mussel shells were used as a source of calcium and phosphoric acid (85 wt% in H_2_O, Sigma Aldrich, Darmstadt, Germany) as a phosphorus source. The calcium carbonate (CaCO_3_) powder obtained from the crushed mussel shells was mixed with a 1 M phosphoric acid (H_3_PO_4_) aqueous solution in a 1.67 Ca/P molar ratio. The synthesis was carried out in a high-energy 3D shaker mixer using zirconia balls (diameter 6 mm) added in a 5:1 ball-to-powder weight ratio. The treatment was carried out for 4 h and then the slurry was dried at 150 °C overnight. An in-depth characterization of the mussel shells and the obtained hydroxyapatite is reported in previous works [47,48,51]. Here, the mussel shell-derived powder was characterised by X-ray diffraction (XRD) on an Italstructures Imaging Plate Diffractometer (IPD3000), equipped with a Co source (Kα = 1.7902 Å) and operating under 30 V and 40 A for the identification of the crystallographic phases. XRD patterns were analysed by Rietveld refinement using the software package MAUD^®^ 2.999. In this work, synthetic hydroxyapatite (HAPs, Ca_5_(OH)(PO_4_)_3_, nanopowder, <200 nm particle size (BET), Sigma Aldrich, Darmstadt, Germany) was used for comparison. The size and the morphology of the powders were analysed by using a field-emission scanning electron microscope (FE-SEM, Zeiss Supra 40, Carl Zeiss, Oberkochen, Germany). The powders were coated with a thin layer of Pt/Pd before the analysis with the FE-SEM.

The zeta potential of the HAPm and HAPs particles (n = 3) was measured at a pH = 5.5–6 at 37 °C with a ZetaSizer Pro (Malvern Panalytical Ltd., Malvern, UK).

HAPm and HAPs nanoparticles dispersed in the SilMA solution were observed with a scanning transmission electron microscope (S-TEM), ThermoFisher TALOS F200S (Thermo Fisher Scientific Inc., Waltham, MA, USA), at a maximum electron voltage of 200 kV. The samples were prepared by pouring a drop of Sil-MA solution with HAP particles in different concentrations (0.5–1.0–2.0 wt%) on a carbon film-supported copper TEM grid and allowed to air dry before observation.

#### 2.1.3. Sponges Preparation

Hydroxyapatite powders were previously dissolved in distilled water and sonicated using a Hielscher Ultrasound UP400S three times for 15 s at 100% amplitude. A LAP stock solution was prepared by dissolving LAP (2,4,6-trimethylbenzoyl)phosphinate (LAP, C_16_H_16_LiO_3_P, TCI America, Portland, OR, USA) in distilled water at a concentration of 40 mg/mL. Then, the components were mixed with the SilMA aqueous solution with the compositions reported in Table 1.

**Figure 1:**
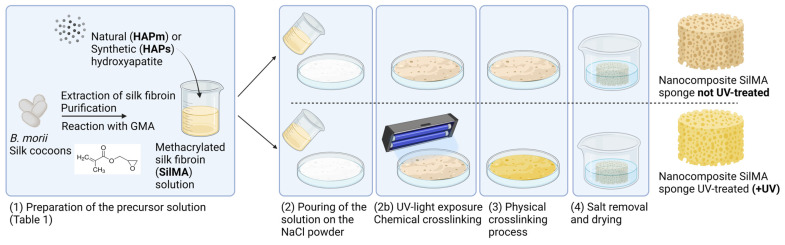
Schematic of the nanocomposite SilMA sponge manufacturing process. Created with BioRender.

Porous SilMA sponges were obtained from a combination of solvent casting and salt leaching methods exploiting the physical crosslinking of the structure, adapting a previously reported protocol [53]. Moreover, some conditions were further treated with a UV-photocrosslinking process for the chemical crosslinking of the structure in the presence of the photoinitiator LAP. A schematic of the process is shown in Figure 1.

Sieved sodium chloride (NaCl, Sigma-Aldrich) with a particle diameter in the range 300–500 μm was placed in a Petri dish. Then, the SilMA solutions reported in Table 1 were slowly poured onto the salt. Half of the samples (referred as UV-treated samples) were left out to set for 20 min and then exposed to UV light (SpotLED curing equipment at 365 nm) for 2 min to complete the chemical crosslinking. 

Consequently, all the samples were left at room temperature for 3 days to allow for SilMA gelation. The sponges were then washed with deionized water for 4 days to remove the excess salt. The samples were cut in cylindrical shapes (diameter 8 mm) with a biopsy punch, air dried and sterilised by autoclaving at 121 °C for 15 min.

### 2.2. Material Characterization 

#### 2.2.1. Microstructural Analysis and Porosity Evaluation

The analysis of the microstructure and the porosity was performed by field emission scanning electron microscopy. Samples were sputtered with a thin Pt/Pd coating and observed with a field-emission scanning electron microscope (FE-SEM, Zeiss Supra 40, Carl Zeiss, Germany) at 4 kV in secondary electron mode. The size of the pores was analysed with ImageJ software 2017 (NIH, Stapleton, NY, USA), measuring around 100 pore diameters for each of the tested conditions. Pore distribution was analysed assuming a Gaussian distribution of pore dimension using OriginPro 2018 and expressed as mean ± standard error of the mean.

#### 2.2.2. Water Absorption

The water absorption capacity of the sponges was evaluated by monitoring the weight of the samples in PBS over 72 h and tested at 30 min, 1 h, 2 h, 24 h, 48 h and 72 h. The initial dry weight was measured (W_0_), and then the samples were incubated in PBS at 37 °C. At each timepoint, samples were removed from the PBS solution. The excess solvent was removed with filter paper, and the wet weight was recorded (W_1_). The swelling ratio was determined as the difference between the wet and dry weights normalised by the dry weight, as reported in Equation (1):

SR = (W_1_ − W_0_)/W_0_ (1)

#### 2.2.3. Compression Test

The mechanical properties of sponges were determined by unconfined uniaxial compressive tests using an Instron^®^ 5969 testing machine equipped with a 10 N load cell. The samples were incubated in PBS for 24 h at 37 °C to reach equilibrium swelling. The tests were carried out using a zero-stress initial condition. Cylindrically shaped sponges were compressed at a constant speed (3 mm/min). The diameter and thickness of each sample were measured before the test with a digital calliper. At least n = 4 samples were used for the measurements. The slope within the initial linear region of the stress–strain plot was used to calculate the Young’s modulus of each sponge. All data are represented as means ± standard deviation (st. dev.). 

### 2.3. Preliminary In Vitro Evaluation

#### 2.3.1. Cell Culture and Seeding

The cytocompatibility of the sponges was tested with MG63 cells (human osteosarcoma cell lines). Cells were cultured in Minimum Essential Medium (MEM, Euroclone, Pero, Italy) supplemented with 10% *v*/*v* fetal bovine serum (FBS, Euroclone, Italy), 1% *v*/*v* antibiotic–antimycotic 100×, 1 mM non-essential amino acid, 2 mM L-glutamine and 1 mM sodium pyruvate. MG63 were cultured as monolayers at 37 °C and 5% CO_2_, changing the complete medium every 2 days. Upon reaching 80% confluency, the cells were detached with trypsin (3 min at 37 °C), and the suspension was centrifuged for 5 min at 1200 rpm, resuspended and seeded on the sponges at a concentration of 20,000 cells/sponge.

#### 2.3.2. AlamarBlue Assay

An AlamarBlue assay was used to analyse cell metabolism at different time points (days 1, 3 and 7 after seeding). The analysis was performed at each timepoint by incubating the samples and the blank controls (sponges without the cells) with resazurin reagent (C_12_H_7_NO_4_, Chemodex Ltd., St. Gallen, Switzerland) at a concentration of 10% in complete medium for 3 h at 37 °C and 5% *v*/*v* CO_2_. After the incubation time, 100 µL of solution was collected from each well, transferred in a black 96-well plate and measured with a plate reader (Tecan Infinite M200) with an excitation wavelength of 535 nm and an emission wavelength of 590 nm. After the reading, the samples were washed three times with PBS, then complete medium was added to the well and the samples were incubated at 37 °C for the next reading since the test was not destructive. Measurements are displayed as means ± st. dev. The statistical analysis of cell proliferation data was performed with a two-way analysis of variance (ANOVA). Bonferroni’s multiple comparison test was used to evaluate the significant differences among the tested conditions. All analyses were performed using OriginPRO. *p*-values were set at four different significance levels: *p* < 0.05 (* *p* ≤ 0.05, ** *p* ≤ 0.01, *** *p* ≤ 0.001, **** *p* ≤ 0.0001).

#### 2.3.3. Cell Morphology

The morphology of the cells seeded on the sponges was evaluated by scanning electron microscopy (FE-SEM, Zeiss Supra 40, Carl Zeiss, Germany). Samples were analysed at each timepoint (days 1, 3 and 7 after seeding) in duplicates. At each timepoint, the samples were fixed with a cacodylate buffer solution of 0.4 M, then incubated at 4 °C for 30 min and then washed three times with a cacodylate buffer solution of 0.2 M. Then, the samples were washed with increasing concentrations of ethanol solution and air dried under the fume hood. After drying, samples were sputter coated with Pt/Pd and analysed using FE-SEM at 4 kV.

## 3. Results and Discussion

### 3.1. Hydroxyapatite Nanoparticle Characterization

**Figure 2:**
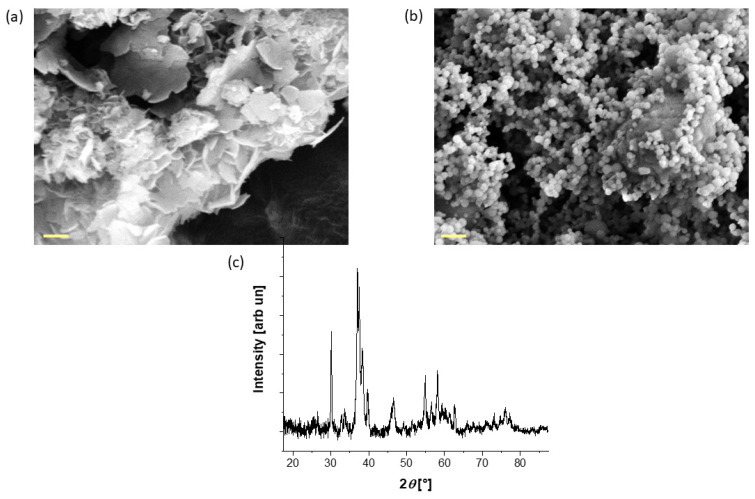
FE-SEM images of the (**a**) as-synthesised mussel shell-derived hydroxyapatite and (**b**) the commercial hydroxyapatite used in the sponge production; scale bar: 200 nm. (**c**) XRD pattern of the mussel shell-derived hydroxyapatite.

In this work, hydroxyapatite from two different sources (natural and synthetic) has been used. Figure 2a,b shows the morphology of the particles, displaying a remarkable difference in shape. The natural-derived hydroxyapatite (HAPm) has a nanometric flake morphology, with a length of up to 900 nm and a thickness of up to a few nm, while the synthetic hydroxyapatite (HAPs) is fully round-shaped with a diameter of less than 200 nm. 

Moreover, since the production process of the natural-based hydroxyapatite particles requires a conversion from calcium carbonate to nanocrystalline hydroxyapatite, an XRD analysis was performed to assess the conversion. The XRD pattern reported in Figure 2c confirms the presence of pure hydroxyapatite in accordance with previous studies [51], with a crystallite size equal to 44 nm.

In the design of the suspension containing HAPm and HAPs, the zeta-potential of the particles was evaluated at pH = 6, corresponding to the pH of the SilMA solution at 37 °C. The zeta-potential values of HAPm and HAPs were determined to be negative, measuring −16.18 ± 0.32 mV and −8.98 ± 0.81 mV, respectively, in alignment with previous studies [54,55]. 

Both HAPm and HAPs were negatively charged within the range of 0 to −20 mV, indicative of a state of “highly unstable” or “relatively stable” colloidal dispersion [56].

Consequently, to optimise the filler concentration within the sponges, various concentrations of nanoparticles were evaluated when dispersed in the SilMA solution, and their dispersion features were assessed using scanning transmission electron microscopy (S-TEM). A limitation in the SilMA suspension development is the impossibility of sonicating directly the protein suspension, since the process is known to trigger beta-sheet formation [57]. 

**Figure 3:**
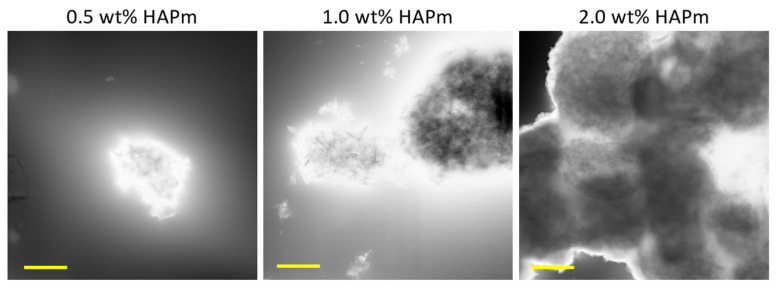
S-TEM images of the HAPm nanoparticles dispersed in SilMA solution at three different concentrations (0.5–1.0–2.0 wt%); scale bar: 2 µm.

Analysis of S-TEM images (Figure 3) revealed that at a concentration of 2.0 wt%, the particles exhibited excessive agglomeration, whereas at 1.0 wt% and 0.5 wt%, agglomerates were present but limited to dimensions lower than 5 µm. Therefore, 1.0 wt% HAP was chosen as the reference concentration for this study.

### 3.2. Morphology and Porosity Evaluation

Methacrylated-silk fibroin (SilMA) sponges with and without the inclusion of hydroxyapatite nanoparticles were prepared by a combination of physical and chemical methods, i.e., salt leaching and UV-photocrosslinking, respectively. The tested formulations and crosslinking conditions are reported in Table 1. 

**Figure 4:**
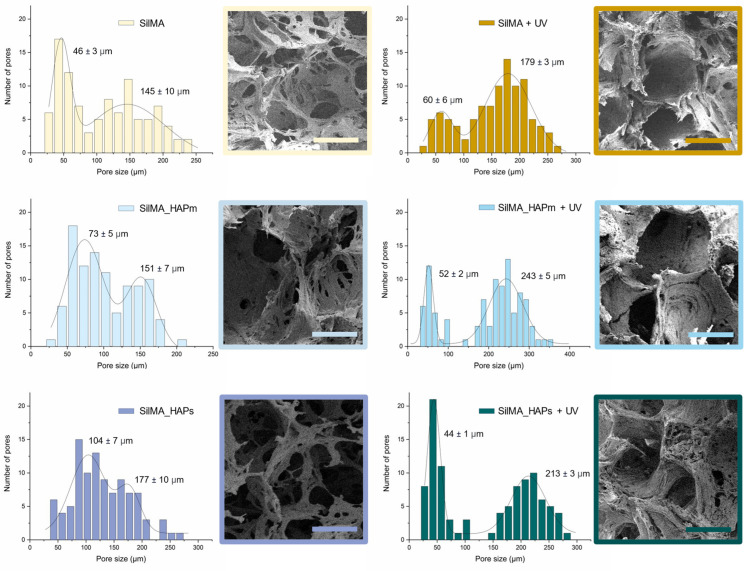
FE-SEM images of the internal porous structure of the dry SilMA sponges and the pore size bimodal distributions measured from FE-SEM images in both non-UV treated and UV-treated conditions; scale bar: 100 µm.

The morphology of the sponges’ inner structure was investigated by scanning electron microscopy. SEM images reported in Figure 4 displayed a highly porous structure for all the tested samples. Clusters of hydroxyapatite nanoparticles were not detectable, proving that a good dispersion of the solid particles in the SilMA solution was achieved (as depicted in Figure 3). The displayed pores were mostly round-shaped with a high degree of interconnectivity. As depicted in Figure 4, a bimodal distribution of the pores was observed under all conditions, with the smaller pores distributed on the surface of the bigger pores. This allows for the formation of an interconnected network, which is fundamental in the design of tissue-engineered scaffolds [7,58].

From the analysis of the pore size, the diameter of the smaller pores was found to be in the range of 30 to 100 µm, while the bigger pores were in the range of 120–300 µm. Generally, the UV-photocrosslinked samples, especially the two with natural or synthetic hydroxyapatite, displayed slightly larger pores and a more organised structure than the controls. This might be an effect of the stabilisation of the scaffolds before the leaching of the salt. 

The pore size of the SilMA sponges studied in this work is promising for their use in bone regeneration since the presence of a bimodal distribution of pores can favour cell responses [59,60]. 

Generally, pore sizes in the range of 200–500 µm are considered optimal for osteoblast proliferation and to promote a good exchange of nutrients and the formation of vascular networks, while around 50 µm pores are optimal for the initial cell adhesion and short-term proliferation in vitro [61]. The pore size achieved with the double-crosslinked SilMA sponges is within this favourable range of pore sizes.

### 3.3. Water Absorption Test

**Figure 5:**
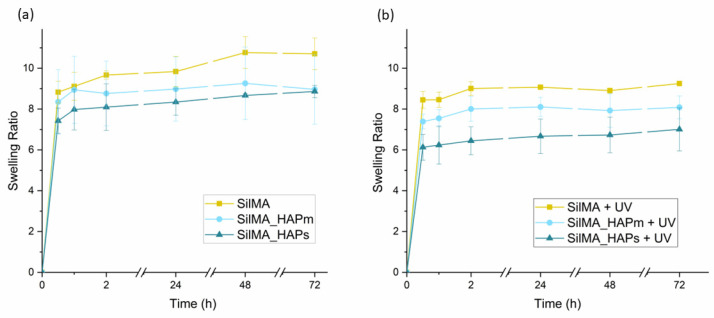
Water uptake curves of the sponges, both non-UV treated (**a**) and UV-treated (**b**), incubated in PBS at 37 °C. Data are represented as means with standard deviations (n = 3).

The curves shown in Figure 5 represent the water uptake capacity of the tested sponges. This test was performed in order to understand the ability of the SilMA scaffolds to uptake liquids, which is an important feature for the bone scaffolds. The scaffolds should guarantee a humid environment for cells and permit the transport of nutrients and the removal of waste. All the conditions displayed similar swelling profiles. The UV-treated samples (Figure 5b) displayed a lower uptake of water compared to the non-treated ones (Figure 5a), and this can be attributed to the presence of additional crosslinking in this set of samples [62]. After 24 h, all the samples reached the plateau. Thus, this time was considered the equilibrium swelling time. After 48 h, some of the non-UV-treated samples were difficult to handle due to excessive swelling and small cracks in the material. Among these, the samples with the hydroxyapatite were especially prone to cracks that could be due to low bonding adhesion among the matrix components. 

### 3.4. Compression Test

**Figure 6:**
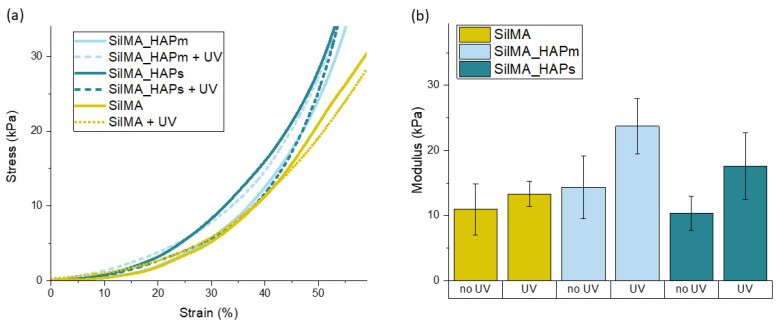
(**a**) Representative stress–strain curves for the tested sponges with and without the UV treatment. (**b**) Compressive elastic moduli (in kPa) of the tested sponges measured after 24 h of incubation in PBS (pH = 7.4) at 37 °C presented as means with standard deviations (n = 4).

The mechanical properties of the SilMA sponges were evaluated by determining the compressive elastic moduli from the stress–strain curves obtained by carrying out unconfined uniaxial compression tests. The obtained compressive moduli were used to compare the mechanical properties of the different sets of samples. Specifically, eventual differences by varying either the compositions (with or without hydroxyapatite) or the crosslinking method (UV-treated or not) were investigated. The results obtained from the mechanical compression tests are displayed in Figure 6. All the tested conditions showed similar behaviour of the stress–strain curves. The elastic moduli, analysed by fitting the first linear region of the curve, were found to be in the range of 10 to 30 kPa, and considering the concentration of SilMA used, they are in accordance with values reported in other studies [63].

Moreover, as shown in Figure 6B, there is a slight difference in the mechanical properties of the samples that underwent a UV treatment with respect to the controls. It was observed that for all the tested compositions, the mean values of the compressive moduli of the UV-treated samples were higher than the nontreated samples. This difference can be correlated to the presence of both physical and chemical crosslinking, highlighting the positive effect of photocrosslinking on improving the properties of the material. This increase in the modulus due to the UV treatment was previously reported [35], with values higher than what was found in this work. The discrepancy in the absolute values of the compressive moduli might be correlated to the different test conditions and parameters, the concentration of SilMA, the linear region analysed for the calculation of the modulus and the manufacturing process [64].

The mechanical properties of the tested sponges are significantly lower than the mechanical properties of bone. However, they can be strategically used as temporary supports to fill bone defects, promoting cellular growth in the early stage of bone remodelling, for example, in the soft callus formation. Soft materials have been reported to be beneficial in this stage for promoting cell activity and the deposition of their ECM [5]. Notably, the tested SilMA-based sponges demonstrate an elastic modulus comparable to that of the osteoid matrix (~35 kPa) [65], a precursor of bone tissue. Furthermore, it has been demonstrated that substrates having elastic moduli within the range of 25–40 kPa promote an upregulation of osteocalcin expression and induce mesenchymal stem cell differentiation towards osteoblasts [66]. Alternatively, SF-based soft materials with good biological properties can be used as fillers in combination with any load-bearing material, i.e., titanium lattice structures [67].

### 3.5. Preliminary In Vitro Biological Test

**Figure 7:**
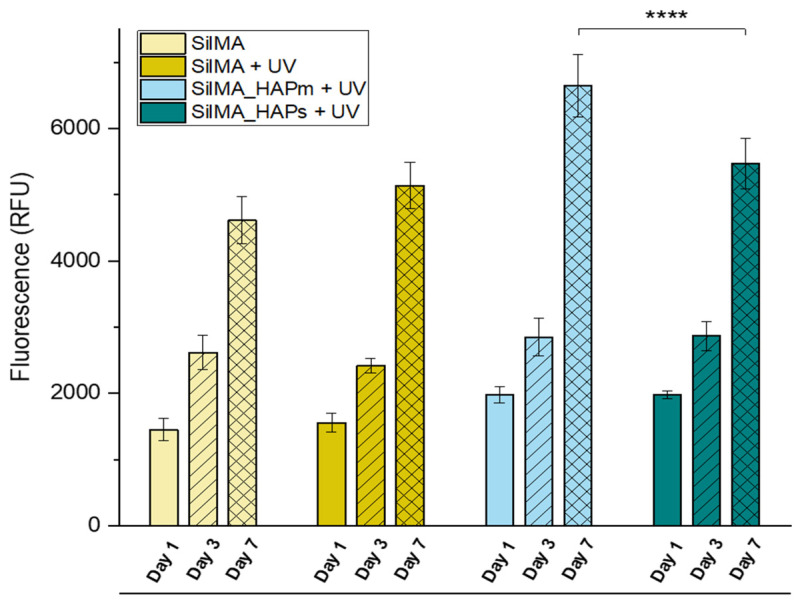
AlamarBlue assay measured over 7 days of MG63 culture on the UV-treated SilMA sponges (SilMA + UV), either with mussel-shells derived hydroxyapatite (SilMA_HAPm + UV), or with synthetic hydroxyapatite (SilMA_HAPs + UV) sponges and the non-UV treated sponge (SilMA). **** *p* < 0.0001.

The evaluation of a preliminary in vitro cytocompatibility test was carried out on the sponges by analysing the viability of the MG63 osteosarcoma cell line. For this study, only the SilMA sponge and the UV-treated set of samples were tested due to the instability of the non-UV-treated nanocomposite sponges during the 7-day time frame. Cells were seeded on top of the samples, and cell adhesion was investigated by scanning electron microscopy, while metabolic proliferation activity was studied by an AlamarBlue assay (Figure 7). As shown in Figure 7, all the conditions displayed metabolically active cells during all the tested time frames (up to 7 days). In particular, an increase in the cell metabolic activity of all the samples was detected in this time frame; noticeable differences were observed from day 1 to day 3 and day 7 for all the tested conditions. Interestingly, a statistically significant increase in metabolic activity was found in the sample with the natural-derived hydroxyapatite at day 7 compared to the synthetic one and the controls. This can be correlated to the presence of organic residues or trace elements in the particles (like Mg, K, Sr and Na), which have been reported to facilitate cell adhesion and the spreading of stem cells [51]. These results suggest an increased cellular proliferation capacity in the presence of natural-derived hydroxyapatite particles, which promotes their use in bone tissue engineering scaffolds.

**Figure 8:**
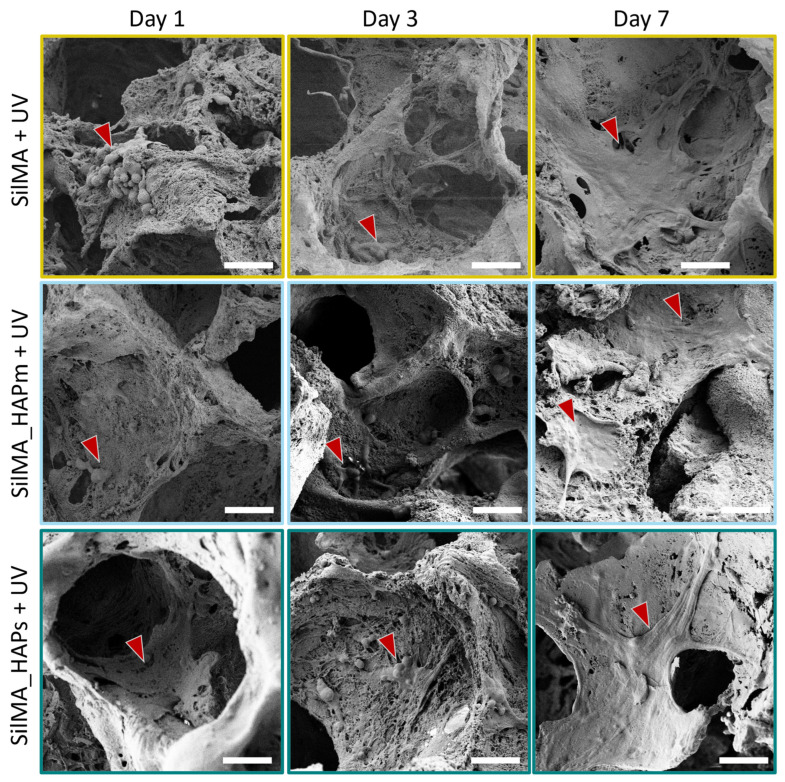
Scanning electron microscope images at day 1, day 3 and day 7, representative of the cells seeded on the UV-treated sponges: SilMA + UV (SilMA only), SilMA_HAPm + UV (with mussel-shells derived hydroxyapatite) and SilMA_HAPs + UV (with synthetic hydroxyapatite). Scale bar: 50 µm. Red arrows indicate cells and cellular morphology details.

To supplement the AlamarBlue assay, scanning electron microscopy was used to evaluate the morphology of the cells seeded on the scaffolds. Some representative images for the UV-treated conditions are shown in Figure 8. At day 1, MG63 cells were mostly round-shaped and organised in clusters under all conditions. However, a few and short protrusions formed, showing an initial adhesion to the samples. After three days, the clusters were still far from each other, but the cell shape became more elongated, with protrusions covering large surfaces of the sample. On day 7, all the cells were mostly spread and stretched on the sample surface, covering entire areas of the samples and showcasing better cell–cell and cell-scaffold interactions. Overall, in all the tested conditions, there was a noticeable increase in the number of cells adhered to the surfaces from day 1 to day 7. Moreover, the shape of the cells changed over time, from more rounded-shaped cells organised in a few clusters far from each other (at day 1) to well-stretched cells exhibiting cell-scaffold interactions and covering most surfaces of the samples at day 7. This, in addition to the proliferation assay, confirms their suitability for bone tissue engineering. 

The aim of this preliminary in vitro evaluation was to investigate the cell viability and their ability to adhere to and proliferate on the scaffolds that were produced. In conclusion, the samples displayed their suitability by favouring cell proliferation and adhesion in all the tested conditions, with better cell proliferation observed in natural-derived hydroxyapatite scaffolds. 

Further in-depth analysis may help in better informing the importance of natural-derived hydroxyapatite in these scaffolds. For example, osteogenic differentiation of bone marrow derived mesenchymal stem cells or inclusion of patient-derived samples can further inform the extensive use of such scaffolds with different cell models.

## 4. Conclusions

The present study highlights the potential of using methacrylated silk fibroin (SilMA) sponges as versatile scaffolds for bone tissue engineering. Through a combination of physical and chemical methods, i.e., salt leaching and UV-photocrosslinking, respectively, the properties of the scaffolds were tailored to meet the requirements for the development of bone scaffolds. 

Incorporating hydroxyapatite nanoparticles, whether synthetic or natural-derived, enhanced the scaffold’s bioactivity and mechanical integrity. The characterization of SilMA sponges’ properties, such as porosity analysis, water absorption and mechanical tests, provides evidence of the effect of different fabrication parameters and the presence or absence of the bioactive components. Furthermore, the assessment of the osteosarcoma cell behaviour on the scaffolds displays their potential for bone defect repair applications.

Overall, our results suggest that double-crosslinked SilMA-based scaffolds, particularly when augmented with natural-derived hydroxyapatite, represent promising candidates for biomimetic scaffold development tailored to specific needs for bone defect repair. Further investigation could delve into exploring other sources of natural-derived hydroxyapatite, optimizing the fabrication parameters and conducting more specific in vitro studies to assess the bone regeneration potential of the studied scaffolds.

## Figures and Tables

**Table 1:** Composition of the SilMA sponges tested in this work.

Sample	SilMA* [% *w*/*v*]	HAPm* [% *w*/*v*]	HAPs* [% *w*/*v*]	LAP* [% *w*/*v*]	UV-Treatment
SilMA	7	-	-	-	No
SilMA_HAPm	7	1	-	-	No
SilMA_HAPs	7	-	1	-	No
SilMA + UV	7	-	-	0.5	Yes
SilMA_HAPm + UV	7	1	-	0.5	Yes
SilMA_HAPs + UV	7	-	1	0.5	Yes

* SilMA: methacrylated silk fibroin; HAPm: mussel shell-derived hydroxyapatite; HAPs: synthetic hydroxyapatite; LAP: 2,4,6-trimethylbenzoyl)phosphinate.

## Data Availability

Data is available from the corresponding author upon reasonable request.
